# Glutamate to GABA ratio is elevated in patients with IDH-mutant lower-grade gliomas and seizures

**DOI:** 10.1093/noajnl/vdaf155

**Published:** 2025-08-20

**Authors:** Riccardo Pascuzzo, Roberta Rudà, Peter B Barker, Jianwei Xiang, Luigi Antelmi, Ruben Gianeri, Alessia Pellerino, Francesca Mo, Riccardo Soffietti, Alberto Bizzi

**Affiliations:** Neuroradiology Unit, Fondazione IRCCS Istituto Neurologico Carlo Besta, Milan, Italy; Division of Neuro-Oncology, Department of Neuroscience, University of Turin, Turin, Italy; Russell H. Morgan Department of Radiology and Radiological Science, Johns Hopkins University School of Medicine, Baltimore, Maryland, USA; Russell H. Morgan Department of Radiology and Radiological Science, Johns Hopkins University School of Medicine, Baltimore, Maryland, USA; Neuroradiology Unit, Fondazione IRCCS Istituto Neurologico Carlo Besta, Milan, Italy; Neuroradiology Unit, Fondazione IRCCS Istituto Neurologico Carlo Besta, Milan, Italy; Division of Neuro-Oncology, Department of Neuroscience, University of Turin, Turin, Italy; Division of Neuro-Oncology, Department of Neuroscience, University of Turin, Turin, Italy; Division of Neuro-Oncology, Department of Neuroscience, University of Turin, Turin, Italy; Neuroradiology Unit, Fondazione IRCCS Istituto Neurologico Carlo Besta, Milan, Italy

**Keywords:** epilepsy, IDH-mutant gliomas, glutamate, GABA, magnetic resonance spectroscopy

## Abstract

**Background:**

Patients with IDH-mutant gliomas often experience seizures that significantly affect their quality of life and outcome. Seizure activity may be the result of dysregulation of excitatory (glutamate, Glu) and inhibitory (gamma-aminobutyric acid, GABA) neurotransmitters in peritumoral tissue. A non-invasive measurement of Glu (in combination with glutamine, termed Glx) and GABA is feasible with proton magnetic resonance spectroscopy (^1^H-MRS). The aim of this study was to determine whether IDH-mutant glioma patients with seizures exhibit altered Glx and GABA levels compared to patients without seizures.

**Methods:**

We conducted a prospective study involving 23 glioma patients (15 with, 8 without seizures), who underwent single-voxel ^1^H-MRS using the MEGA-PRESS sequence. ^1^H-MRS data were collected from volumes of tissues in the tumor/peritumoral regions and parietal cortex used as control. Metabolite ratios (Glx/Cr, GABA/Cr, Glx/GABA) were analyzed and correlated with seizure presence and other clinical-pathological parameters. Longitudinal ^1^H-MRS data in a subset of 10 patients were also acquired.

**Results:**

At first study, the Glx/GABA ratio was significantly higher in the tumor/peritumoral tissue of patients with seizures compared to those without (*P* = .023). Longitudinal data confirmed this finding, showing consistently elevated Glx/GABA values in patients with seizures. Moreover, patients taking two or more antiseizure medications had significantly higher Glx/GABA ratios and lower GABA/Cr ratios in the peritumoral region.

**Conclusion:**

Glioma patients with seizures have an altered balance of Glx and GABA in tumor/peritumoral tissue, supporting the hypothesis that neurotransmitter imbalances contribute to seizure activity. ^1^H-MRS may provide non-invasive biomarkers for identifying neurotransmitter dysregulation in glioma-related epilepsy.

**Key Points**

- In the tumor/peritumoral tissue of patients with seizures, Glx/GABA ratios were elevated at the first study and confirmed at follow-up.
- Patients on two or more antiseizure medications had higher Glx/GABA and lower GABA/Cr ratios, suggesting drug resistance may be linked to neurotransmitter imbalance.

**Importance of the Study**

This study supports the hypothesis that a significant imbalance between excitatory and inhibitory neurotransmitters (ie, elevated Glx/GABA ratios) in the peritumoral tissue of patients with IDH-mutant glioma is associated with seizure activity. These results align with those of prior ex-vivo studies. Furthermore, the study underscores the relevance of GABA levels in glioma-related epilepsy and their relationship to antiseizure medication efficacy. These insights suggest that ^1^H-MRS is a valuable tool for monitoring neurotransmitter dysregulation in glioma patients, with the potential of guiding personalized treatment strategies. Future research should focus on larger cohorts and explore the utility of ^1^H-MRS in optimizing antiepileptic therapy, enhancing the management of seizure-related morbidity in glioma patients.

In the 2021 WHO classification adult-type diffuse gliomas are classified based on both traditional histological features and molecular characteristics (isocitrate dehydrogenase [IDH1 or IDH2] mutations and 1p/19q codeletion).^1^ Lower-grade gliomas (ie, WHO grade 2 or 3) include IDH1- or IDH2-mutant, 1p/19q-codeleted oligodendrogliomas, and IDH1- or IDH2-mutant astrocytomas.

Surgical resection is the most important therapeutic option, and the role of adjuvant radiotherapy and chemotherapy in high-risk patients is well established.^2–5^ Recently, vorasidenib, an IDH1 and IDH2 inhibitor, has been shown to significantly increase MRI-based progression-free survival and delay time to next intervention in non-enhancing grade 2 gliomas after surgery.^6^

Seizure is the most common symptom in patients with IDH-mutant gliomas, causing significant morbidity and impacting quality of life. Glioma patients are also often resistant to antiseizure medications (ASMs).^7^ Several factors may increase the risk of seizures, including age, tumor volume, location, histotype, and molecular characteristics.^8–10^ The presence of altered neurotransmitter concentrations in peritumoral tissue, in particular the excitatory neurotransmitter glutamate (Glu), is hypothesized to be a major factor favoring seizure activity. Glioma cells release high levels of Glu^11^ and glutamate receptors have been shown to be over-expressed in glioma cells and peritumoral astrocytes.^12^ In a mouse tumor model, the finding of abnormalities in γ-aminobutyric acid (GABA) concentrations and GABAergic disinhibition in peritumoral tissue also supports that they may further enhance epileptogenicity.^13,14^

The concentration of the combined signal of Glu and glutamine (Gln), usually abbreviated as “Glx,” can be measured in vivo using proton magnetic resonance spectroscopy (^1^H-MRS). Glx can be measured with conventional ^1^H-MRS methods such as the PRESS sequence; however, GABA, due to its relatively low concentration, is harder to measure using this approach. The “MEGA-PRESS” ^1^H-MRS sequence^15^ is an advanced spectral editing methodology that can estimate GABA with greater precision than the conventional PRESS sequence, while also providing a reasonable estimate of Glx.^16^ In the literature, there have been only a few MRS studies that have investigated the concentration of Glu or Glx in patients with brain tumors and seizures.^17–19^ Results have been somewhat inconsistent, most likely reflecting differences in methodology and/or patient selection.

This prospective study was designed to test the hypothesis that seizure activity results from an imbalance between excitatory (ie, Glx) and inhibitory (ie, GABA) neurotransmitters in the tumor/peritumoral tissue. The main aim of the study was to determine whether there are significant differences in Glx and GABA between patients with and without seizures. Thus, Glx and GABA levels were measured using the MEGA-PRESS single-voxel ^1^H-MRS in patients with residual grade 2 and 3 IDH-mutant glioma after surgery. MRS data were acquired from the tumor/peritumoral areas and from a control area in the parietal cortex, remote from the tumor. Longitudinal ^1^H-MRS assessment was performed in a subset of patients. The relationships of Glx and GABA at first ^1^H-MRS and follow-up visits of each patient were evaluated and correlated with seizure activity and several clinical and histo-molecular factors.

## Patients and Methods

### Patients and Clinical Assessment

In this prospective study, patients referred to the Division of Neuro-Oncology, University of Turin, who met the following inclusion criteria, were recruited:

- age ≥ 18 years;
- grade 2 or 3 IDH-mutant astrocytomas and IDH-mutant 1p/19q-codeleted oligodendrogliomas according to WHO 2021;
- presence or absence of residual tumor after surgery assessed with volumetric MRI;
- presence or absence of seizures.

All patients underwent at least one MRS study. In addition, ten patients had one or more follow-up MRS studies. A clinical and neurological examination at the time of each MRS study was performed. Tumor type (astrocytoma vs oligodendroglioma) and grade (2 vs 3), tumor site (temporo-insular vs other), extent of surgical resection (gross total vs subtotal/partial), time from neurosurgical procedure to first MRS study (< 2 years, ≥ 2 years), and epilepsy duration before the first MRS study (< 3 years vs ≥ 3 years) were categorized. At the time of each visit the following factors related to epilepsy were evaluated and categorized: seizure frequency; seizure type (focal aware, focal with impaired awareness, generalized according to ILAE classification)^20^; type (drugs acting on the GABAergic system or not) and number of ASM (≤ 1 vs ≥ 2 drugs); concomitant radiotherapy and/or chemotherapy (yes or no) and disease status (progression, stable disease, partial response, complete response) according to Response Assessment in Neuro-Oncology (RANO) criteria.^21^ Patients were divided into three groups according to seizure frequency: low seizure frequency (1–10 seizures per month), intermediate seizure frequency (11–30 seizures per month), and high seizure frequency (≥ 30 seizures per month).

Patients gave informed consent to the procedures, which followed the Declaration of Helsinki for human experiments and were approved by the Ethical Committee of the Fondazione IRCCS Besta Institute, Milan, Italy.

### Magnetic Resonance Spectroscopy

**Figure 1.**
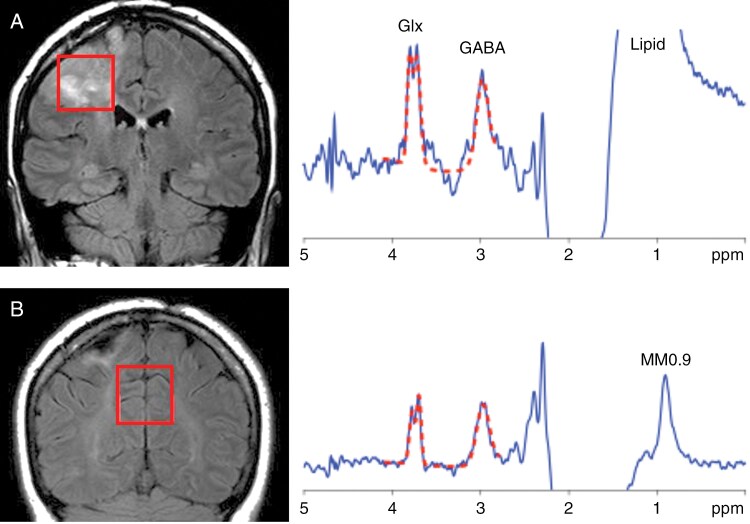
Example voxel locations (red box) and MEGA-PRESS spectra from a 40 y.o female with a right frontal oligodendroglioma and 3–5 seizures per month, in (A) tumor and (B) uninvolved parietal lobe. Peaks are identified from glutamate plus glutamine (Glx), GABA, and a macromolecular peak resonating at 0.9 ppm (MM0.9). In addition, the tumor spectrum shows some lipid contamination from adjacent peri-cranial fat. The spectral data (blue lines) are overlaid (dashed red lines) from 4.2 to 2.8 ppm with the model fit result from the “Gannet” software package. The Glx/GABA ratio in the tumor measures ≈ 1.3, and it is higher than in the bilateral parietal cortex (≈ 0.8) remote from the tumor and used as a control.

All scans were performed on a Philips (Best, The Netherlands) 3T “Achieva” scanner equipped with a 32-channel receive head coil. After conventional anatomical brain imaging was performed, levels of GABA and Glx were estimated using the MEGA-PRESS spectral editing sequence.^22^ Scan parameters were 3 × 3 × 3 cm voxel size, TR/TE 2000/68 ms, 320 excitations (160 with a 14 ms editing pulse applied at 1.9ppm, 160 with it applied at 7.5 ppm), giving a scan time of 10 min 40s per voxel. CHESS water suppression (50 Hz bandwidth) was used. Prior to acquisition, field homogeneity was optimized up to second order using a FASTMAP^23^-based routine. In addition to the water-suppressed scans, 8 excitations were recorded without water suppression. Two different volumes of interest (VOI) were acquired in each patient with voxel placement guided by a senior neuroradiologist (AB): one VOI including tumor and peritumoral tissue and a “control VOI” positioned in the biparietal gray matter remote from the tumor that was assumed to be free of tumor infiltration. The aim was to place the voxel at the interface of tumor with peritumoral tissue, that is believed to be the most likely epileptogenic area, while avoiding the resection cavity, the cerebrospinal fluid, bone, and foci of prior hemorrhage likely to cause susceptibility artifacts. An example of the VOI placement and the corresponding edited MR spectra from one patient with seizures is shown in Figure 1.

Data were analyzed using the program “Gannet 3.1.2.”^24^ After phase- and frequency-correction applied to individual excitations,^25^ sum and difference spectra were calculated. GABA and Glx were estimated by parametric curve fitting of the difference spectra as described previously,^24^ and creatine (Cr) was estimated from the sum spectra. Three ratios were calculated for each brain region, GABA/Cr, Glx/Cr, and Glx/GABA.

### Statistical Analysis

Wilcoxon’s rank-sum test was used to investigate differences in MRS metabolite ratios (Glx/GABA, Glx/Cr, and GABA/Cr) in the peritumoral area between patients with or without seizure.

Available follow-up data were included in the analysis using a linear mixed-effects regression model, which specified a patient-specific random effect for grouping the repeated measures of the same patient. The following fixed effects were included and tested one at the time in the aforementioned model: presence of seizures (yes or no), tumor location (temporo-insular or another area), 1p/19q codeletion (yes vs no), use of ASM acting on the GABAergic system (yes vs no), number of ASM (≤ 1 or ≥ 2), extent of tumor resection (gross total vs subtotal/partial), time from last neurosurgical intervention to MRS examination (< 2 years vs ≥ 2 years), and concomitant radiotherapy and/or chemotherapy (yes vs no). Multiple factors showing *P* < .10 at univariable analysis were entered as fixed factors in a multiple mixed-effects regression model (with patient-specific random effect) to provide a more comprehensive picture of the relationships between these factors and the metabolite ratios, adjusting for confounders and determining the independent contributions of each factor. This analysis was also performed on the metabolite ratios measured in the parietal (control) region.

In the subgroup of patients with seizures, other linear mixed-effects regression models were used to test whether differences in frequency, type, and duration of seizures were associated with changes in the metabolite ratios measured in the tumor/peritumoral area.

Intra-patient variability of the three ratios was computed in the parietal and tumor/peritumoral area for each subject with longitudinal data available using the coefficient of variation (CV), defined as the ratio of the standard deviation to the mean value of a patient. Inter-patient variability was estimated by computing the CV between patients, considering the mean value of each patient when follow-up data were acquired. In addition, we tested whether there were significant changes in metabolite ratios over time in patients with seizures by fitting a linear mixed-effects model with a patient-specific random intercept and considering the time from first MRS examination as fixed effect.

Statistical analysis was performed in R (version 3.6.0), and “lme4” and “lmerTest” packages were used for the mixed-effects models.^26,27^

## Results

### Patient Characteristics

**Figure 2.**
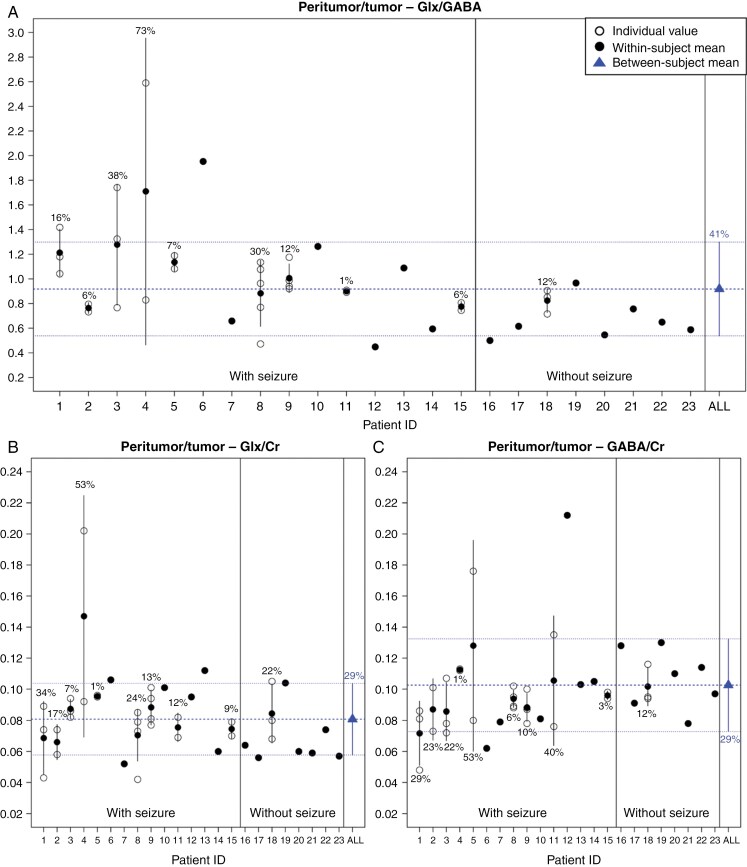
Within-subject mean and individual values of Glx/GABA (A), Glx/Cr (B), and GABA/Cr (C) in the peritumor/tumor region are illustrated separating subjects with and without seizures. The between-subject mean value is plotted in blue. Error bars represent ± one standard deviation from the mean. The intra-subject variability is expressed as the coefficient of variation (CV), reported as percentage for each subject with multiple studies. The inter-subject variability is expressed as the between-subject CV, in blue.

Twenty-four patients were enrolled in this study, 12 males and 12 females (median age at baseline evaluation of 42.7 ± 6.7 years, range 30–54 years). Flowchart of patient selection is illustrated in Supplementary Figure S1. One patient was excluded from the analysis due to MRS technical failure secondary to presumed head motion in tumor/peritumoral VOI. Demographic, clinical, and neuropathological data of the 23 patients included in the analysis are reported in Table 1. The majority of patients (17/23, 73.9%) were treated with levetiracetam (Keppra), either as monotherapy or in combination with other ASMs (Supplementary Table S1). Metabolite ratios of the 23 patients are illustrated in Figure 2.

**Table 1.** Demographic, Clinical, and Neuropathological Characteristics of Patients With and Without Seizures.

Characteristic	Patients with seizure (*n* = 15)	Patients without seizure (*n* = 8)
**Age at baseline evaluation, years (mean ± SD)**	44.3 ± 6.3	40.1 ± 6.9
**Sex**		
Male	7	4
Female	8	4
**Time from symptom onset, years (mean ± SD)**	5.4 ± 4.8	5.9 ± 3.7
**Number of follow-up MRS studies**		
0	6	7
1	5	-
2	2	1
3	1	-
4	1	-
**Follow-up period, months (mean ± SD)**	17.4 ± 9.5	21.5
**Tumor location**		
Temporo-insular	10	2
Other (frontal and/or parietal)	5	6
**Time from surgical intervention to baseline MRS examination** ^*^		
< 2 years	6	2
≥ 2 years	8	5
**Glioma tumor classification** ^**^		
Grade 2, IDH mutant, 1p/19q codeleted	5	6
Grade 3, IDH mutant, 1p/19q codeleted	2	1
Grade 2, IDH mutant, 1p/19q intact	6	1
Grade 3, IDH mutant, 1p/19q intact	1	-
**Extent of resection** ^***^		
Gross total	6	2
Subtotal or partial	8	5
**Disease status**		
Stable disease	11	5
Tumor progression	4	3
**Use of drugs acting on the GABAergic system**		
Yes	5	1
No	10	7
**Number of antiseizure medications**		
0–1	4	5
2–3	11	3
**Concomitant chemotherapy**		
Yes	2	2
No	13	6
**Seizure frequency per month**		
1–10	8	-
11–30	5	-
>30	2	-
**Seizure type**		
Focal aware	10	-
Focal with impaired awareness	4	-
Generalized	1	-

Abbreviations: SD = standard deviation; IDH = isocitrate dehydrogenase; MRS = magnetic resonance spectroscopy.

^*^The MRS examination was performed only before the neurosurgical intervention in one patient with seizure, and data on the intervention were not available in one patient without seizure.

^**^Neuropathological results on tumor were not available in one patient with seizure.

^***^Extent of resection was not available in one patient with seizure and in one without seizure.

At the time of the first ^1^H-MRS study, 15 patients had seizures while 8 did not. Eight of the 15 patients had less than five seizures per month, in 5 patients the frequency was between 10 and 30, and in 2 patients it was above 30 (ie, 90 seizures per month). Seizure type was focal aware in 10 patients, focal with impaired awareness in 4, and generalized (primary or secondary) in 1. Tumor progression was observed in 26.7% (4/15) of patients with seizures and 37.5% (3/8) of those without, with no significant difference between the two groups (Fisher test, *P* = .657). Longitudinal data were available in 10 patients with an average time period of 17.8 ± 9.0 months (range 6.5–31.1). Longitudinal data were available in 9 patients with seizures: 6 patients had seizures at all MRI examination points, while 3 were free from seizures at their second and last MRI. Among the 8 patients without seizures, longitudinal data were available in 1 patient, who remained seizure-free.

### Variation of Metabolite Ratios with Respect to Clinical-pathological Data

At first ^1^H-MRS study, Glx/GABA values were significantly higher (*P* = .023) in the tumor/peritumoral area of patients with seizures (1.019 ± 0.378) compared to those without seizures (0.691 ± 0.170; *P* = .023) (Table 2). No other metabolite ratios in the tumor/peritumoral area showed significant differences between the two groups.

**Table 2.** Comparison of Metabolite Ratios (Glx/GABA, Glx/Cr, and GABA/Cr) in the Peritumoral Area of Patients With or Without Seizure, at First MRS Evaluation.

Metabolite ratio	Patients with seizures (*n* = 15)	Patients without seizures (*n* = 8)	*P*-value
Glx/GABA	1.019 ± 0.378	0.691 ± 0.170	**.023**
Glx/Cr	0.083 ± 0.021	0.072 ± 0.021	.333
GABA/Cr	0.103 ± 0.043	0.108 ± 0.018	.245

Data are represented as mean ± standard deviation. Significant differences (*P*-values < .05) are indicated in bold.

Abbreviations: Cr = creatine; Glx = glutamate and glutamine.

Similar results were obtained by including follow-up ^1^H-MRS measurements in the mixed-effects model (Table 3) that accounted for the dependency of the data acquired from the same patient: on average, patients with seizures had Glx/GABA values in the tumor/peritumoral area greater by 0.323 (95% CI 0.074–0.573, *P* = .0056) compared to those without seizures; in addition, higher Glx/Cr values were observed in patients with seizures (*P* = .0312). As for the other factors, we found that patients taking 2 or more drugs instead of 0 or 1 had increased Glx/GABA ratio in the tumor/peritumoral area by 0.281 (0.001–0.561, *P* = .0270) as well as decreased GABA/Cr ratio by 0.039 (−0.058 to −0.020, *P* < .0001).

**Table 3.** Results of Mixed-effects Models for Metabolite Ratios in Tumor/peritumoral Area.

Factor	Group	Glx/GABA		Glx/Cr		GABA/Cr
		*Univariable LM* *β (95% CI, p)*	*Multiple LM* *β (95% CI, p)*	*Univariable LM* *β (95% CI, p)*	*Multiple LM* *β (95% CI, p)*	*Univariable LM* *β (95% CI, p)*
Seizure	Absent	ref	ref	ref	ref	ref
	Present	**0.345 (0.094 to 0.596, *P* = .0035)**	**0.323 (0.074 to 0.573, *P* = .0056)**	0.015 (-0.003 to 0.032, *P* = .0532)	**0.017 (0.001 to 0.033, *P* = .0312)**	-0.002 (-0.022 to 0.018, *P* = .4332)
Tumor location	Temporo- insular	ref	-	ref	-	ref
	Other	0.113 (-0.165 to 0.391, *P* = .2129)	-	0.006 (-0.012 to 0.025, *P* = .2461)	-	-0.007 (-0.029 to 0.014, *P* = .2479)
1p/19q codeletion	Absent	ref	-	ref	-	ref
	Present	0.138 (-0.141 to 0.417, *P* = .1665)	-	0.010 (-0.009 to 0.029, *P* = .1572)	-	0.009 (-0.014 to 0.032, *P* = .2150)
Number of ASM	0–1	ref	ref	ref	-	ref
	2–3	**0.312 (0.015 to 0.608, *P* = .0196)**	**0.281 (0.001 to 0.561, *P* = .0270)**	0.003 (-0.017 to 0.024, *P* = .3716)	-	**-0.039 (-0.058 to -0.020, *P* < .0001)**
Use of drugs acting on the GABAergic system	No	Ref	-	ref	-	ref
	Yes	0.006 (-0.288 to 0.300, *P* = .4852)	-	0.005 (-0.015 to 0.025, *P* = .3022)	-	-0.002 (-0.025 to 0.020, *P* = .4155)
Concomitant radio- chemotherapy	No	ref	ref	ref	ref	ref
	Yes	-0.239 (-0.552 to 0.074, *P* = .0674)	-0.071 (-0.368 to 0.226, *P* = .3197)	-0.014 (-0.034 to 0.006, *P* = .0803)	-0.006 (-0.026 to 0.014, *P* = .2718)	-0.005 (-0.027 to 0.017, *P* = .3395)
Time from surgery	< 2 years	ref	-	ref	-	ref
	≥ 2 years	-0.058 (-0.343 to 0.227, *P* = .3443)	-	0.002 (-0.016 to 0.020, *P* = .4124)	-	0.008 (-0.011 to 0.026, *P* = .2155)
Extent of resection	Gross total	ref	ref	ref	ref	ref
	Partial	**0.230 (0.003 to 0.457, *P* = .0434)**	**0.266 (0.040 to 0.493, *P* = .0107)**	0.015 (-0.004 to 0.034, *P* = .0580)	0.014 (-0.005 to 0.032, *P* = .0739)	0.006 (-0.016 to 0.029, *P* = .2948)

Effects of the seizure presence and of other clinico-pathological factors are reported for each metabolite ratio measured in the tumor area. Reported numbers are regression (*β*) coefficients (with 95% CI and *P*-value) of the mixed-effects model including only one factor at the time (univariable LM) and of the mixed-effects model including those factors that showed *P* < .10 at univariable analysis (multiple LM). Significant results (*P* < .05) are in bold. Abbreviations: VOI = volume of interest; LM = linear mixed-effects model; ASM = antiseizure medication; CI = confidence interval; ref = reference level.

Moreover, when the extent of tumor resection was partial, the Glx/GABA ratio values in the tumor/peritumoral area were increased by 0.266 (0.040–0.493, *P* = .0107) with respect to gross total tumor resection.

The presence of seizures was significantly associated with a slight increase in the Glx/Cr ratios (0.005, 95% CI 0.001–0.010, *P* = .0249) measured in the parasagittal bilateral parietal gray matter that was acquired as a control measurement (Supplementary Table S2). Moreover, the use of drugs influencing the GABAergic system determined a significant decrease in the GABA/Cr ratio of this control region (−0.011, 95% CI −0.021 to −0.002, *P* = .0094) (Supplementary Table S2), as well as a significant increase in the Glx/GABA ratio (0.094, 0.013–0.176, *P* = .0114). Moreover, patients with 1p/19q-codeleted tumors had lower GABA/Cr ratio (−0.009, −0.018 to −0.000, *P* = .0247) and higher Glx/GABA ratio in the parietal region (0.077, 0.002–0.152, *P* = .0362) than 1p/19q-intact tumors (Supplementary Table S2). No other variables had significant associations with the metabolite ratios measured in this control area.

In the cohort of 15 glioma patients with seizures, variations of metabolite ratios in the tumor/peritumoral VOI were not significantly associated with type, duration, and frequency of seizures.

### Variability of Metabolite Ratios Over Time

**Figure 3.**
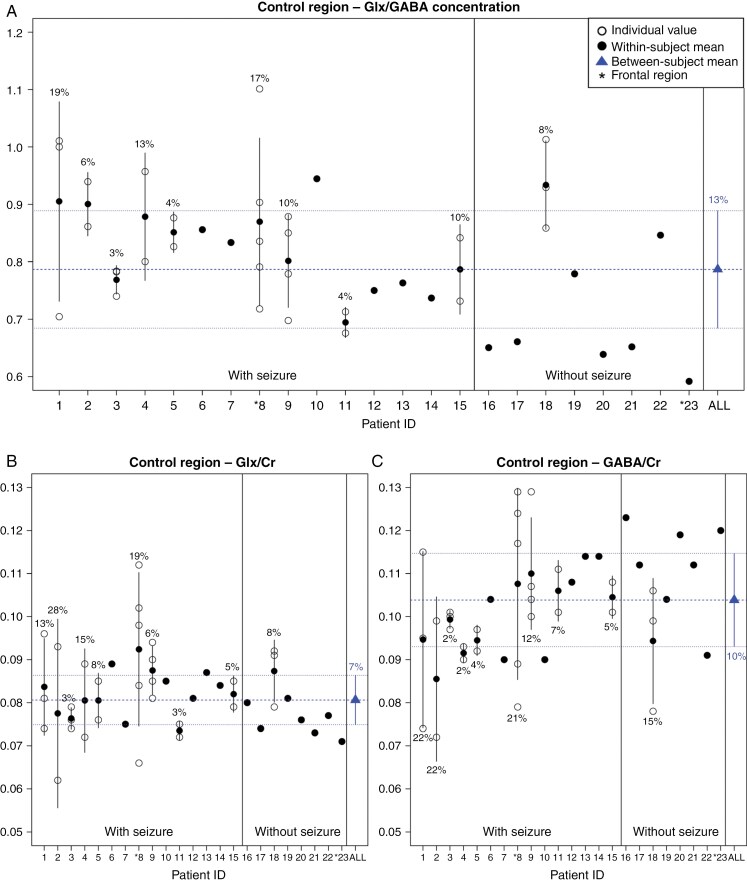
Within-subject mean and individual values of Glx/GABA (A), Glx/Cr (B), and GABA/Cr (C) in the control region (ie, parietal cortex) are illustrated separating subjects with and without seizures. In two patients (indicated with an asterisk), the control measures were obtained from the frontal region because tumor was in the parietal region. The between-subject mean value is plotted in blue. Error bars represent ± one standard deviation from the mean. The intra-subject variability is expressed as the coefficient of variation (CV), reported as percentage for each subject with multiple studies. The inter-subject variability is expressed as the between-subject CV, in blue.

All metabolite ratios in the parietal gray matter had lower average within-patient CV than in tumor/peritumoral VOI (Supplementary Table S3), suggesting that the intra-patient variability over time was higher in tumor/peritumoral areas than in the biparietal parasagittal gray matter. Inter-patient variability of the metabolite ratios was, on average, greater than the corresponding intra-patient CV in tumor/peritumoral VOI (Supplementary Table S3), while they were similar in the parietal gray matter VOI (Figure 3). These results may in part be explained by the greater homogeneity of tissue specimens in the parietal cortex compared with tumor/peritumoral tissue.

In the subgroup of 9 patients with seizures who had longitudinal data available, no significant variations of the three metabolite ratios were observed over time (Supplementary Figure S2).

## Discussion

This study used in-vivo ^1^H-MRS study to evaluate Glx and GABA levels in glioma patients with or without seizures, and correlated alterations of these metabolites with seizure parameters such as semeiology, duration, type, and number of ASM, concomitant antineoplastic treatments, and pathological factors (ie, tumor location, 1p/19q codeletion). The main result was that patients with seizure activity had higher Glx/GABA ratios in the tumor/peritumoral tissue at their first ^1^H-MRS examination. This key result supports the hypothesis that seizure activity is associated with an imbalance between Glu and GABA neurotransmitters in the tumor/peritumoral tissue and is in agreement with ex-vivo results obtained on surgical samples.^11,28^ No other metabolite ratios (ie, Glx/Cr, or GABA/Cr) showed significant differences between the two groups of patients at the first ^1^H-MRS examination.

Furthermore, clinical and ^1^H-MRS follow-up examinations were performed in a subgroup of patients, with the aim to assess metabolite changes over time. The higher Glx/GABA values in tumor/peritumoral tissue of patients with seizures became more consistent and robust in the follow-up, and Glx/Cr values became significantly higher in patients with seizures, likely due to an increase in statistical power.

A lower GABA/Cr ratio, and consequently a higher Glx/GABA ratio, were also observed in patients taking 2 or more ASMs in comparison with those taking only one drug or not taking ASM. This is consistent with the concept that patients with drug-resistant epilepsy may continue to have a lower concentration of GABA in the tumor/peritumoral tissue.

Several studies have been carried out to evaluate the role of Glu and GABA in epilepsy. The first studies were published in the 1990s and used ex-vivo ^1^H-MRS to determine levels of metabolites in brain specimens from patients with intractable epilepsy. In one study,^29^ increased GABA levels were found in biopsy specimens from patients with tuberous sclerosis complex and mild cortical dysplasia; however, increased GABA levels did not provide protection from seizures and did not correlate with antiepileptic treatment. Subsequent studies used ^1^H-MRS to evaluate in vivo levels of GABA and Glu (or Glx) in patients with refractory epilepsy. One study^30^ found elevated levels of Cho, Glx, Glx/tNAA, and GABA+/Cr ratios in 15 patients with epilepsy associated with malformations of cortical development compared to 15 healthy controls. Another study revealed an increase in Glx and Glx/NAA ratio measured with ^1^H-MRS in the frontal lobe of patients with idiopathic generalized epilepsy; however, no alterations in GABA levels were found.^31^ In a series of 10 patients with Sturge-Weber syndrome, ^1^H-MRS detected higher Glu/Cr ratios in the affected hemisphere in comparison with the contralateral one, and a strong positive correlation with seizure frequency was found.^32^

Concerning brain tumors, ^1^H-MRS has been historically used to evaluate peaks of Cho, NAA, creatine, lactate, lipids, and their ratios, mainly for differential diagnosis between gliomas, lymphomas, metastases, and other non-neoplastic diseases of the CNS. More recently, the potential role of ^1^H-MRS in detecting 2-hydroxyglutarate (2-HG), the product of IDH-1 or -2 mutations in gliomas, was reported.^33–36^ To date, there are few studies exploring the role of ^1^H-MRS in tumor epileptogenesis and evaluating brain neurotransmitters in peritumoral tissue. In a series of 13 patients, it was found that a higher Glx/Cr ratio existed in the peritumoral tissue.^37^

Recently, Neal and colleagues^17^ used 7T magnetic resonance imaging to quantify the concentration of Glu and other metabolites in the tumor and peritumoral area of 10 patients with grade 2 and 3 gliomas with seizures and compared Glu levels with clinical variables like tumor aggressiveness and epilepsy. They applied both *glutamate chemical exchange saturation transfer* (GluCEST), a glutamate-weighted imaging method, and single-voxel ^1^H-MRS. Increased peritumoral GluCEST contrast was found to be associated with both recent seizures (occurring within a month before MRI evaluation) and drug-refractory epilepsy. Furthermore, a positive correlation between GluCEST contrast in tumor/peritumoral voxels and Glu measured with ^1^H-MRS was found; however, no correlations between ^1^H-MRS glutamate and seizure characteristics were reported.

There are some limitations of the current study, including the relatively small number of patients accrued. Also due to the low concentration of GABA in the brain, ^1^H-MRS data were acquired from a large (3 × 3 × 3 cm) VOI with constrained geometry that included brain tumor, peritumoral tissue with infiltrating glioma, edema, and some volume of normal-appearing brain tissue. The same expert neuroradiologist was always at the console to position the VOI with the aim of optimizing the balance between pathological tissue with T_2_-FLAIR signal hyperintensity and the peritumoral tissue without MR signal abnormality. Also, since the “on” and “off” editing pulses were placed at 1.9 and 7.5 ppm, it is well known that a macromolecule (with coupled resonances at 1.7 and 3.0 ppm) will also co-edit with the GABA signal, so the “GABA” results reported here are the sum of GABA and MM concentrations which in the literature is often labeled as “GABA+.” Alternative editing schemes, which suppress the MM signal, are available. However, they tend to be less robust as they are more sensitive to B_0_ magnetic field drift; thus, we did not use these methods in this study.^38^ Also, no attempt was made to separate Glu from Gln; thus, it is also possible that the changes in Glx reported here may be at least partially due to changes in Gln. It should be noted that some MRS studies of gliomas have found elevated Gln compared to normal brain.^39–41^ Moreover, when considering the ratios of Glx and GABA to creatine (Cr), it should also be considered that Cr may be abnormal in tumor or peri-tumor regions. Finally, the acquisition time was on the order of 10 min per VOI, which is quite long: while all patients were very motivated and cooperative, it cannot be excluded that a few of them moved during the MRS acquisition. However, only 1 patient was excluded due to excessive head motion, which rendered the MEGA-PRESS data unusable.

Further studies are needed to confirm these results and should include MRS evaluation in specific subgroups of patients taking ASM that are active on the GABAergic and/or glutamatergic systems. In this regard, the current results suggest that measurements of Glx and GABA levels before and during treatment with drugs acting either on the glutamatergic or GABAergic system may help to choose the most suitable and effective ASM. Recent developments in high-field (ie, 7 Tesla) MRS imaging have shown that it is possible to map neurotransmitters such as GABA at relatively high spatial resolution, and also to separate Glu from Gln with a high degree of confidence.^39^ This methodology would appear to be ideal for investigating the role of peritumoral metabolites and neurotransmitters in the pathogenesis of seizures.

Finally, the product of IDH mutations is 2-HG, which has a similar structure and may mimic the function of glutamate, could contribute to the genesis of seizures in IDH-mutant grade 2 gliomas.^42^ Future MRS investigations should, therefore, also monitor the levels of 2-HG in patients with seizures.

In conclusion, this prospective in-vivo ^1^H-MRS study, performed at 3 Tesla in 23 patients with grade 2–3 IDH-mutant gliomas shows that Glx/GABA values are higher in the tumor/peritumoral tissue of patients with seizures with respect to patients without seizures. These results agree with ex-vivo results on surgical samples in other studies, suggesting that ^1^H-MRS might be useful in the future to guide and monitor response to therapy with different choices of ASM.

## Supplementary Material

vdaf155_suppl_Supplementary_Materials_1

## Data Availability

Original data generated during this study will be made available upon reasonable request.
