# Supplementary material for: Glutamate to GABA ratio is elevated in patients with IDH-mutant lower-grade gliomas and seizures
Source: Neurooncol Adv. 2025 Aug 20;7(1):vdaf155. doi: 10.1093/noajnl/vdaf155 (PMC12391666; doi:10.1093/noajnl/vdaf155)
Supplement: vdaf155_suppl_Supplementary_Materials_1 [file vdaf155_suppl_supplementary_materials_1.docx]

**Supplementary Materials**

**Supplementary Table S1.** Anti-seizure medication (ASM) regimens in patients with and without seizures.

| **Group** | **Number of ASMs** | **ASMs used (number of patients)** | **Total number of patients** |
| --- | --- | --- | --- |
| **With seizure**  **(n=15)** | 1 | Keppra: 3000 mg (n=1), 1500 mg (n=1), 1000 mg (n=1)  Tolep: 900 mg (n=1) | 4 |
|  | 2 | Keppra 3000 mg + Vimpat 400 mg (n=2)  Keppra 3000 mg + Vimpat 300 mg (n=2)  Keppra 1500 mg + Vimpat 300 mg (n=1)  Keppra 2000 mg + Tegretol 800 mg (n=1)  Keppra 1000 mg + Tegretol 800 mg (n=1)  Keppra 2500 mg + Tolep 1800 mg (n=1)  Depakin 1500 mg + Vimpat 400 mg (n=1)  Tegretol 800 mg + Vimpat 400 mg (n=1) | 10 |
|  | 3 | Keppra 1750 mg + Tolep 600 mg + Fycompa 2 mg (n=1) | 1 |
| **Without seizure**  **(n=8)** | 0 | – | 2 |
|  | 1 | Keppra: 2000 mg (n=1), 1500 mg (n=1), 1000 mg (n=1) | 3 |
|  | 2 | Keppra 3000 mg + Vimpat 300 mg (n=1)  Keppra 2500 mg + Vimpat 200 mg (n=1) Depakin 1300 mg + Vimpat 300 mg (n=1) | 3 |

**Supplementary Table S2.** Results of mixed-effects models for metabolite ratios in parietal (control) region.

| **Factor** | **Group** | **Glx/GABA - Parietal region** | | **Glx/Cr - Parietal region** | **GABA/Cr - Parietal region** | |
| --- | --- | --- | --- | --- | --- | --- |
|  |  | *Univariable LM*  *β (95% CI, p)* | *Multiple LM*  *β (95% CI, p)* | *Univariable LM*  *β (95% CI, p)* | *Univariable LM*  *β (95% CI, p)* | *Multiple LM*  *β (95% CI, p)* |
| Seizure | Absent | ref | ref | ref | ref | - |
|  | Present | **0.065 (-0.011 to 0.141, p=0.0476)** | 0.040 (-0.039 to 0.118, p=0.1619) | **0.005 (0.001 to 0.010, p=0.0249)** | -0.001 (-0.010 to 0.008, p=0.4037) | - |
| Tumor Location | Temporo-insular | ref | - | ref | ref | - |
|  | Other | -0.014 (-0.098 to 0.070, p=0.3727) | - | -0.001 (-0.006 to 0.005, p=0.4154) | 0.000 (-0.009 to 0.009, p=0.4608) | - |
| 1p/19q codeletion | Absent | ref | ref | ref | ref | ref |
|  | Present | **0.073 (-0.008 to 0.154, p=0.0378)** | **0.077 (0.002 to 0.152, p=0.0362)** | 0.003 (-0.003 to 0.008, p=0.1750) | -0.006 (-0.016 to 0.003, p=0.0961) | **-0.009 (-0.018 to -0.000, p=0.0247)** |
| Number of ASM | 0-1 | ref | - | ref | ref | - |
|  | 2-3 | 0.050 (-0.036 to 0.135, p=0.1273) | - | 0.001 (-0.005 to 0.007, p=0.3463) | -0.006 (-0.016 to 0.004, p=0.1340) | - |
| Use of drugs acting on the GABAergic system | No | ref | ref | ref | ref | ref |
|  | Yes | 0.056 (-0.031 to 0.144, p=0.1021) | **0.094 (0.013 to 0.176, p=0.0114)** | -0.001 (-0.006 to 0.005, p=0.4221) | **-0.008 (-0.016 to -0.001, p=0.0438)** | **-0.011 (-0.021 to -0.002, p=0.0094)** |
| Concomitant radio- chemotherapy | No | ref | - | ref | ref | - |
|  | Yes | 0.016 (-0.090 to 0.122, p=0.3843) | - | 0.002 (-0.006 to 0.010, p=0.3351) | 0.004 (-0.009 to 0.017, p=0.2761) | - |
| Time from surgery | < 2 years | ref | - | ref | ref | - |
|  | >= 2 years | -0.034 (-0.113 to 0.045, p=0.1989) | - | -0.003 (-0.009 to 0.003, p=0.1338) | -0.002 (-0.012 to 0.008, p=0.3384) | - |
| Extent of resection | Gross total | ref | - | ref | ref | - |
|  | Partial | 0.047 (-0.038 to 0.133, p=0.1385) | - | 0.001 (-0.005 to 0.007, p=0.3634) | -0.005 (-0.014 to 0.004, p=0.1213) | - |

Effects of the seizure presence and of other clinico-pathological factors are reported for each metabolite ratio measured in the parietal (control) region. Reported numbers are regression (*β*) coefficients (with 95% CI and p-value) of the mixed-effects model including only one factor at the time (univariable LM) and of the mixed-effects model including those factors that showed p<0.10 at univariable analysis (multiple LM). Significant results (p<0.05) are in bold. Abbreviations: VOI = volume of interest; LM = linear mixed-effects model; ASM = antiseizure medication; CI = confidence interval.

**Supplementary Table S3.** Intra- and inter-patient variability of metabolites ratios.

| **Metabolite ratio** | **Average within-patient CV, % (range)** | | **Between-patient CV, %** | |
| --- | --- | --- | --- | --- |
|  | *Tumor* | *Parietal* | *Tumor* | *Parietal* |
| Glx/GABA | 20.0  (1.3 – 72.8) | 8.6  (3.2 – 19.2) | 41.4 | 13.0 |
| Glx/Cr | 19.1  (0.7 – 52.9) | 10.1  (2.9 – 28.3) | 28.5 | 7.1 |
| GABA/Cr | 19.8  (0.6 – 53.0) | 10.1  (2.1 – 22.3) | 29.1 | 10.4 |

Average within-patient and between-patient coefficient of variation (CV) of metabolite ratios in peritumoral and control (parietal lobe) areas are reported.

**
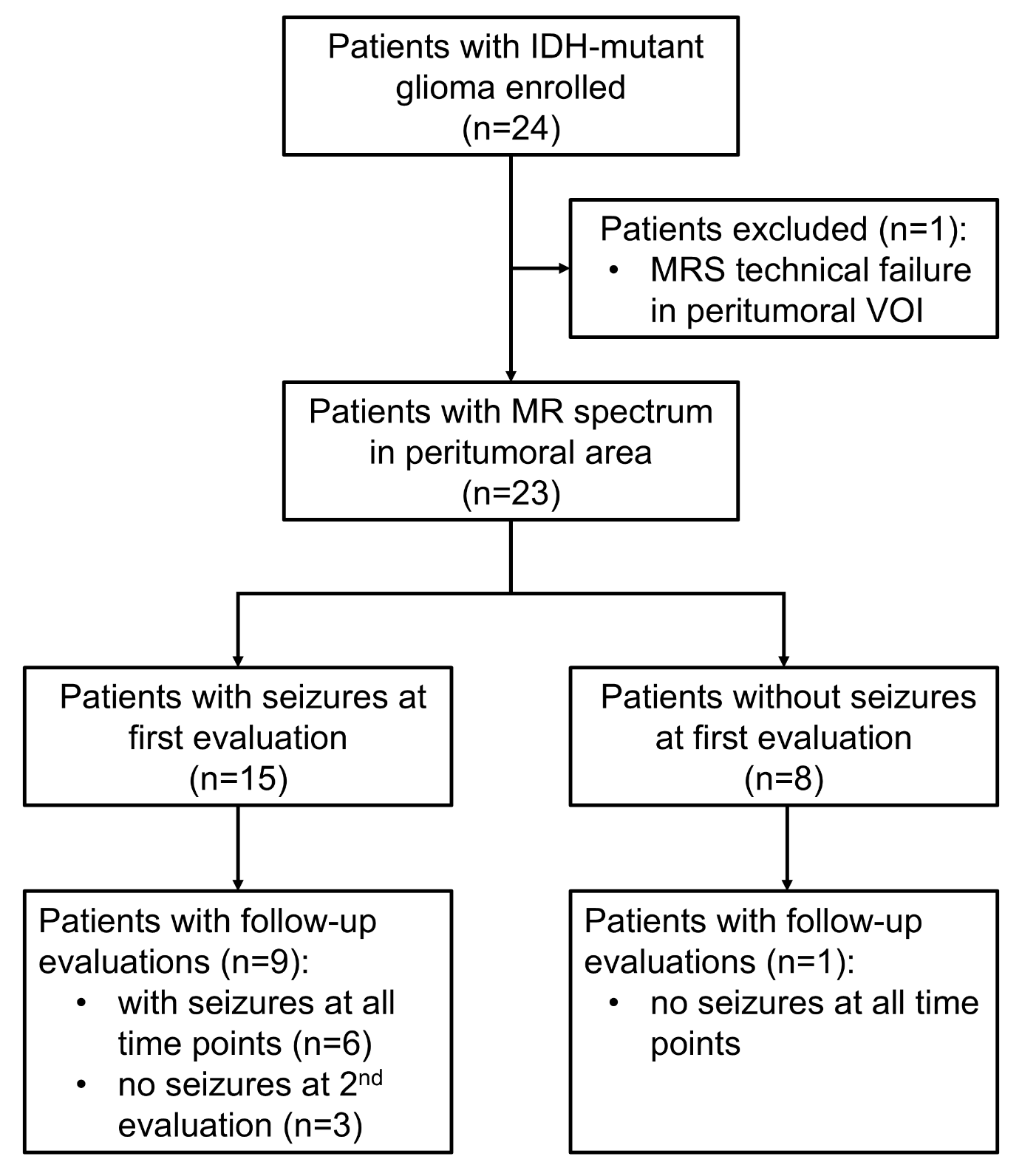
Supplementary Figure S1. Flowchart of patient selection.**

MRS=magnetic resonance spectroscopy; VOI=volume of interest.

**Supplementary Figure S2. Time variation of metabolite ratios in the peritumoral region in the subgroup of patients with seizure.**

**
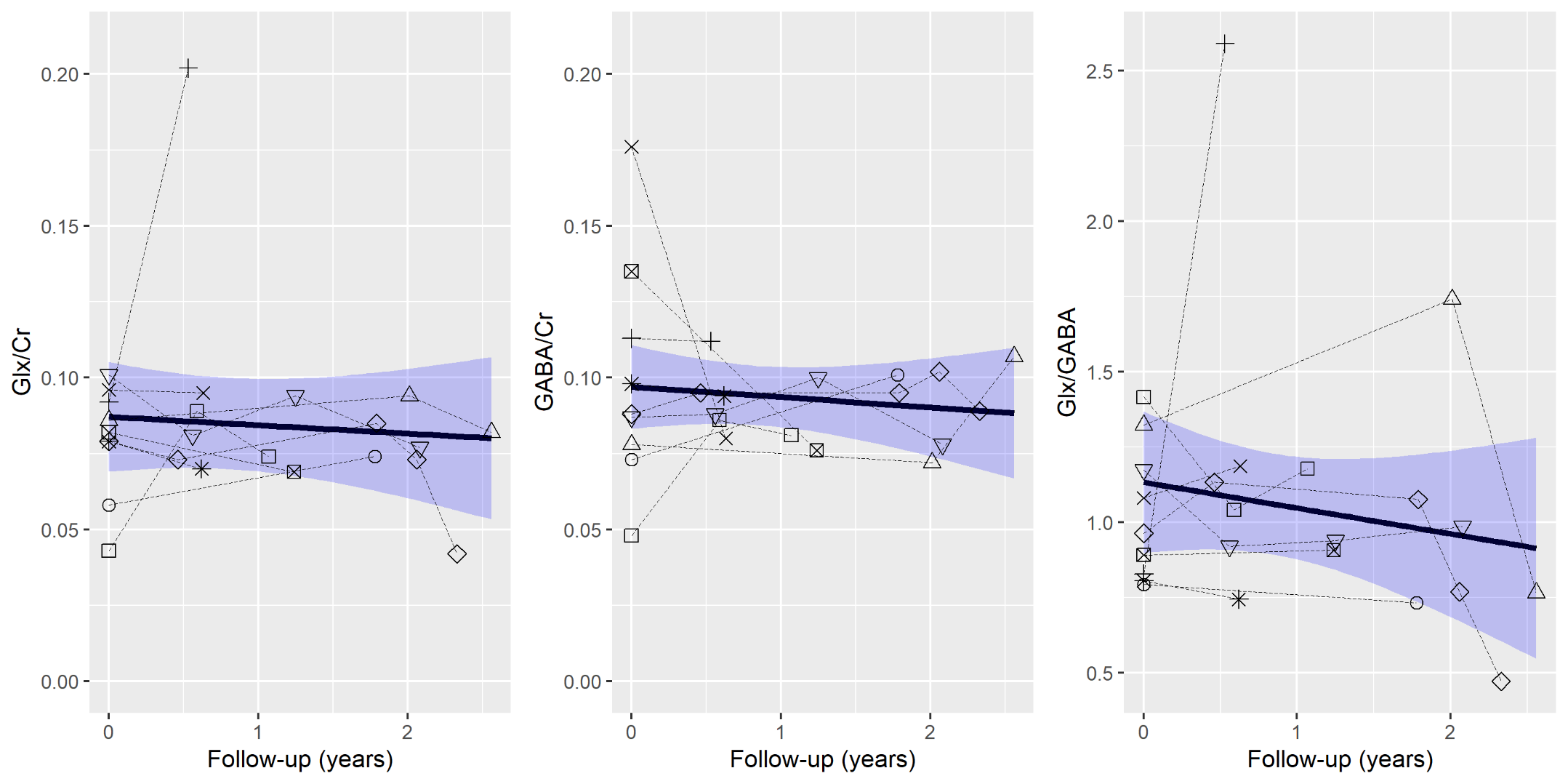
**Each point in the plot represents the metabolite ratio measurement of a patient at one MRS examination. Different point shapes represent different patients, connected with dashed lines to trace individual-level progression. Solid lines represent the fitted linear trend of metabolite ratios over time, as obtained by the linear mixed-effects regression model with the follow-up time considered as fixed effect and with a random patient-specific intercept. Shaded areas represent the 95% confidence interval around the regression line. The time coefficients were nearly zero for all of the three models (Glx/Cr: β [std. err.] = -0.003 [0.006], p=0.666; GABA/Cr: β [std. err.] = 0.003 [0.006], p=0.558; Glx/GABA: β [std. err.] = -0.086 [0.096], p=0.381), indicating no significant overall trends.
